# BAFF promotes follicular helper T cell development and germinal center formation through BR3 signal

**DOI:** 10.1172/jci.insight.183400

**Published:** 2024-11-08

**Authors:** Ye Chen, Maogen Chen, Yu Liu, Qiang Li, Youqiu Xue, Liu Liu, Rongzhen Liang, Yiding Xiong, Jun Zhao, Jingrong Chen, Weidong Lin, Julie Wang, Yun Feng Pan, William Stohl, Song Guo Zheng

**Affiliations:** 1 Department of Rheumatology & Immunology, School of Cell and Gene Therapy, Songjiang Research Institute, Songjiang Hospital Affiliated to Shanghai Jiao Tong University School of Medicine, Shanghai, China.; 2 Division of Rheumatology, Department of Internal Medicine, and; 3 Organ Transplant Center, First Affiliated Hospital of Sun Yat-sen University, Guangzhou, China.; 4 Clinical Research Center, The First Dongguan Affiliated Hospital, Guangdong Medical University, Dongguan, China.; 5 Guangxi Key Laboratory of Tumor Immunology and Microenvironmental Regulation, Guilin Medical University, Guilin, China.; 6 Department of Pharmacy, Shandong Provincial Hospital Affiliated to Shandong First Medical University, Jinan, China.; 7 Division of Rheumatology, Department of Medicine, University of Southern California Keck School of Medicine, Los Angeles, California, USA.

**Keywords:** Autoimmunity, Immunology, Autoimmune diseases

## Abstract

T follicular helper (Tfh) cells represent an important subset of CD4^+^ T cells that is crucial to the maturation and differentiation of B cells and the production of high-affinity antibodies. Because B cell activating–factor (BAFF), a vital B cell survival factor, is also crucial to B cell maturation and differentiation, we assessed the effects of BAFF on Tfh cell development and function. We demonstrated that deficiency of BAFF, but not of APRIL, markedly inhibited Tfh cell development, germinal center (GC) formation, and antigen-specific antibody production. The promoting effect of BAFF on Tfh cell development was dependent on expression of BR3 on T cells, and its promoting effect on GC formation was dependent on expression of BR3 on both T cells and B cells. BAFF directly promoted expression of the Tfh cell–characteristic genes via NF-κB signaling. This effect did need BR3 expression. Thus, BAFF not only has direct effects on B cells, but it also has direct effects on Tfh cell differentiation via engagement of BR3, which collectively promoted GC formation and production of high-affinity antibodies. This dual effect of BAFF on B cells and Tfh cells may help explain the clinical utility of BAFF antagonists in the management of certain autoimmune diseases.

## Abstract 2

BAFF promotes follicular helper T cell development through BR3 on T cells and germinal center formation through BR3 on both T cells and B cells

## Introduction

B cell activating–factor (BAFF), a 285–amino acid member of the TNF superfamily, is best known for its ability to promote B cell survival and differentiation (1). Mature B cells are greatly reduced in mice genetically engineered to be BAFF deficient (2), and pharmacologic neutralization of BAFF in both mice and humans leads to substantial reductions in B cell numbers (3, 4). Closely related to BAFF is APRIL, a 250–amino acid protein member of the TNF superfamily. In contrast to the dramatic reduction of B cells in BAFF-deficient mice, APRIL-deficient mice do not display a reduction in B cells (5, 6).

BAFF has three receptors: BCMA, TACI, and BR3 (7, 8). Of these receptors, BCMA and TACI also bind to APRIL (9–12), whereas BR3 binds only to BAFF (7); BR3 is critical to the survival and maturation of B cells (13), whereas TACI modulates both T cell–independent B cell responses and B cell expansion (14). BCMA has little effect on B cell numbers but is critical to survival of long-lived bone marrow plasma cells and plasmablasts (15, 16), likely through its engagement with APRIL (17).

In addition to the roles played by BAFF and APRIL in B cell numbers and function, follicular helper CD4^+^ T (Tfh) cells provide specific help to follicular B cells (18), are critical for germinal center (GC) formation and maintenance (19), and are vital to the generation of most memory B cells and plasma cells, which produce high-affinity antibodies (20). Of note, BAFF directly stimulates T cell proliferation and cytokine production in vitro (21, 22); affects Th1, Th2, and T regulatory cells in vivo (23, 24); and promotes the Th17 cell–driven disease, experimental autoimmune encephalomyelitis (25). Additionally, blocking BAFF in autoimmune-prone BCMA-deficient B6.*Nba2* mice significantly reduces the numbers of splenic Tfh cells, GC B cells, and plasma cells (26), pointing to an important role for BAFF in maintaining and preserving the number and function of Tfh cells.

A multistage and multifactorial model has been widely accepted for Tfh differentiation (18). DCs provide an initial signal that primes Tfh differentiation in the T cell zone (27), and the precursor Tfh cells then interact with B cells at the T-B border of peripheral lymphoid organs (28). IL-21 and IL-6 play critical roles in Tfh induction (29), and purified CD4^+^ T cells cocultured with APC in the presence of anti-CD3/28, IL-6, IL-21, and anti–TGF-β can be induced into Tfh-like cells that are capable of driving a GC response (30). Because BAFF facilitates DC maturation, promotes IL-6 production, and is essential for follicular DC network formation that initiates Tfh differentiation (31), BAFF may play an important role in Tfh development.

In this study, we used multiple knockout and transgenic mice to analyze the role of the BAFF/APRIL system in the differentiation and development of Tfh cells. Our results highlight the important role of BAFF/BR3 interactions in Tfh cell differentiation. Although B cells facilitate this effect, BAFF can directly act on T cells in the absence of B cells, triggering the NF-κB signaling pathway and, thereby, enhancing expression of BCL-6 and promoting Tfh cell differentiation. These results provide insights into our understanding of the role of BAFF signaling in humoral immunity.

## Results

### In vivo generation of Tfh cells requires BAFF but not APRIL.

**Figure 1:**
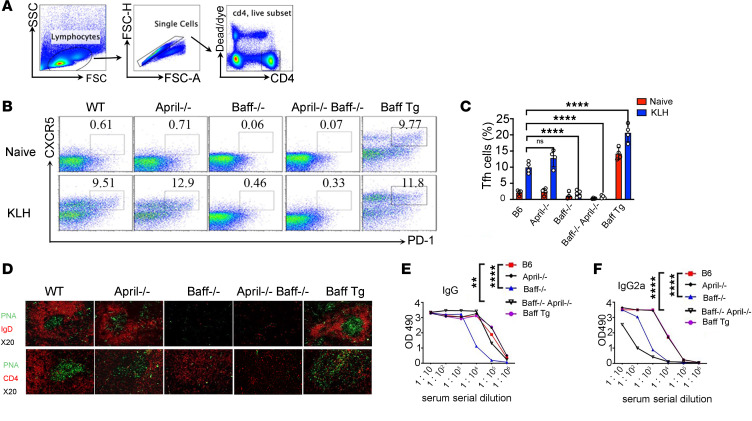
In vivo generation of Tfh cells requires BAFF but not APRIL. WT, *April^–/–^*, *Baff^–/–^*, *April^–/–^*.*Baff*^–/–^, or Baff transgenic mice were immunized with keyhole limpet hemocyanin (KLH) emulsified in complete Freund’s adjuvant (CFA), and mice were euthanized 10 days after immunization. (**A**) Gating strategy for CD4^+^ T cells. (**B** and **C**) The percentages of Tfh cells among CD4^+^ T cells in draining lymph nodes (dLN) were determined by flow cytometry. The representative flow data (**B**) and the statistical data (**C**) are shown. (**D**) Confocal microscopy of germinal centers of LNs. (**E** and **F**) ELISA for KLH-specific IgG and IgG2a in serum (*n* = 3–5 mice per group). Data are presented as mean ± SEM. Data are representative of 3 independent experiments. *P* values were calculated by 1-way ANOVA (**C**) or 2-way ANOVA (**E** and **F**) with a Tukey’s multiple comparison test. For **E**, statistical analysis was performed specifically for the 1:10,000 condition. **P < 0.01, ****P < 0.0001.

As a first step in determining the role of BAFF and APRIL in Tfh differentiation, we analyzed congenic WT, *Baff*^–/–^, *April*^–/–^, *Baff*^–/–^.*April*^–/–^, and *Baff* transgenic (BTg) mice. In the absence of intentional immunization, Tfh cells were expanded in BTg mice, whereas the percentages of Tfh cells in the other analyzed mice were low. In mice immunized with keyhole limpet hemocyanin (KLH) emulsified in complete Freund’s adjuvant (CFA) Tfh responses and GC formation on day 10 after immunization were robust in WT, *April*^–/–^, and BTg mice but not in *Baff*^–/–^ or *Baff*^–/–^.*April*^–/–^ mice (Figure 1, A–C). That is, Tfh cells were constitutively expanded in mice that overexpressed BAFF and were robustly expanded following immunization in hosts that expressed BAFF, regardless of whether APRIL was present.

As Tfh cells are the main cognate helpers of B cells, we assessed GC formation and antibody responses. Neither *Baff*^–/–^ nor *Baff*^–/–^.*April*^–/–^ mice harbored detectable GCs in the draining lymph nodes, whereas GCs were much larger in BTg mice than in WT or *April*^–/–^ mice (Figure 1D). KLH-specific IgG and IgG2a antibody production paralleled the GC findings, with levels in *Baff*^–/–^ and *Baff*^–/–^.*April*^–/–^ mice considerably lower than in WT or *April*^–/–^ mice, with levels in the latter two being identical (Figure 1, E and F). Taken together, these results indicate that BAFF, but not APRIL, is essential to Tfh cell development, GC formation, and antigen-specific antibody production.

### In vivo generation of Tfh cells is dependent on BR3 but independent of BCMA or TACI.

**Figure 2:**
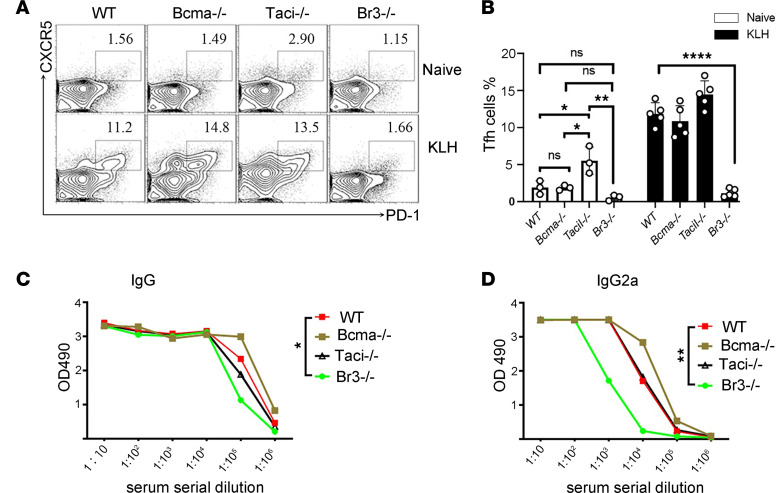
In vivo generation of Tfh cells is dependent on BR3 but independent of BCMA or TACI. WT, *Bcma^–/–^*, *Taci^–/–^*, and *Br3^–/–^* mice were immunized with KLH emulsified in CFA, and mice were euthanized 10 days after immunization. (**A** and **B**) The frequency of Tfh cells among CD4^+^ T cells in dLNs was determined by flow cytometry, and the representative flow data (**A**) and the statistical data (**B**) are shown. (**C** and **D**) ELISA for KLH-specific IgG and IgG2a in serum (*n* = 3–5 mice per group). Data are presented as mean ± SEM. Data are representative of 3 independent experiments. *P* values were calculated by 1-way ANOVA (**B**–**D**) with a Tukey’s multiple comparison test. For **C** and **D**, statistical analysis was performed specifically for the 1:100,000 (**C**) or 1:10,000 (**D**) conditions. *P < 0.05, **P < 0.01, ***P < 0.001, ****P < 0.0001.

To determine the importance of each individual BAFF receptor to Tfh cell development, we first assessed the frequency of Tfh cells in *Taci*^–/–^, *Bcma*^–/–^, and *Br3*^–/–^ mice. Among nonimmunized mice, *Taci*^–/–^ mice harbored a significantly higher percentage of Tfh cells than did WT, *Bcma*^–/–^, or *Br3*^–/–^ mice, with no differences in Tfh cells among WT, *Bcma*^–/–^, or *Br3*^–/–^ mice (Figure 2, A and B). Among immunized mice, *Br3*^–/–^ mice failed to generate a robust Tfh cell response, whereas WT, *Taci*^–/–^ and *Bcma*^–/–^ mice displayed similar robust Tfh cell responses (Figure 2, A and B). In line with the Tfh cell data, levels of KLH-specific IgG and IgG2a antibodies were significantly lower in *Br3*^–/–^ mice than in the other tested mice (Figure 2, C and D). Despite this reduction, BR3^–/–^ KLH-immunized mice still managed to mount a relatively robust IgG response. This observation aligns with those of previous studies (32, 33), suggesting that alternative mechanisms, such as the extrafollicular pathway, may somehow play a role. In the absence of a robust GC response, the extrafollicular pathway could allow B cells to differentiate into antibody-secreting cells outside of GCs, thereby compensating for the impaired GC response and enabling continued antibody production in *BR3^–/–^* mice (34). Collectively, our results suggest that BAFF/BR3 interactions are critical to Tfh differentiation, with APRIL, TACI, and BCMA being dispensable.

### In vivo generation of Tfh cells requires BAFF even with abundance of B cells.

**Figure 3:**
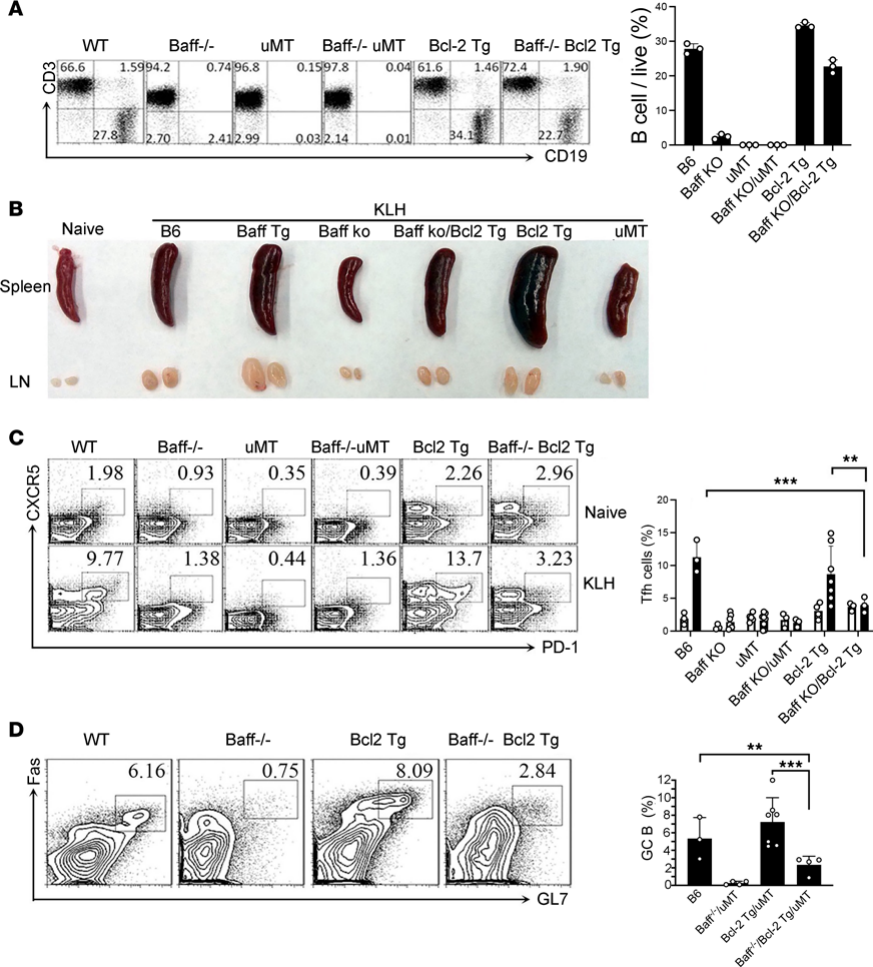
In vivo generation of Tfh cells requires BAFF even with abundance of B cells. (**A**) The percentages of B and T cells in the unimmunized mice (*n* = 3 per group). (**B**) Appearance of spleen and lymphocyte in representative immunized mice. (**C**) WT, *Baff^–/–^*,uMT, *Baff^–/–^* uMT, Bcl-2 Tg, *Baff^–/–^* Bcl2 Tg mice were immunized with KLH/CFA (*n* = 3–7 per group). The frequency of Tfh cells was determined by flow cytometry. The representative flow data (left) and the statistical data (right) are shown. (**D**) The percentages of GC B on B220^+^ cells were determined (*n* = 3–7 per group). Data are presented as mean ± SEM. Data are representative of 3 independent experiments. *P* values were calculated by 1-way ANOVA (**C** and **D**) with a Tukey’s multiple comparison test. **P < 0.01, ***P < 0.001.

Given that B cells contribute to the differentiation of CD4^+^ T cells into Tfh cells (35), we assessed the generation of Tfh cells in B cell–deficient μMT mice and found that Tfh cells were largely absent in both nonimmunized and KLH-immunized mice, leading to smaller spleens and draining lymph nodes (Figure 3, A–C). Not surprisingly, generation of Tfh cells was also largely absent in *Baff*^–/–^.μMT mice, which harbor no B cells (Figure 3, A and C).

Because B cell numbers are markedly diminished in *Baff*^–/–^ mice, the results in μMT mice raised the possibility that the blunted Tfh cell response in *Baff*^–/–^ mice was not directly related to the absence of BAFF but rather to a paucity of B cells. To refute this possibility, we introduced a *Bcl2* Tg into *Baff*^–/–^ mice to generate *Baff*^–/–^.*Bcl2^Tg^* mice. BAFF promotes B cell survival by maintaining the expression of BCL-2 (36), and overexpression of BCL-2 B cells largely restores the numbers, maturation, and function of B cells (37). Indeed, the percentage of B cells in our *Baff*^–/–^.*Bcl2^Tg^* mice was similar to that in WT mice (Figure 3A). The similarity in B cells notwithstanding, immunization of *Baff*^–/–^.*Bcl2^Tg^* mice with KLH failed to augment the percentage of Tfh cells, in sharp contrast to the augmentation observed in WT mice (Figure 3, B and C). Moreover, generation of GC B cells was also blunted in *Baff*^–/–^.*Bcl2^Tg^* mice in KLH-immunized mice (Figure 3D). These findings indicate that BAFF not only regulates B cell homeostasis, but has a direct role in maintaining Tfh cell responses under normal in vivo conditions.

### BAFF engagement with BR3 on T cells is required for generation of Tfh cells in vivo.

**Figure 4:**
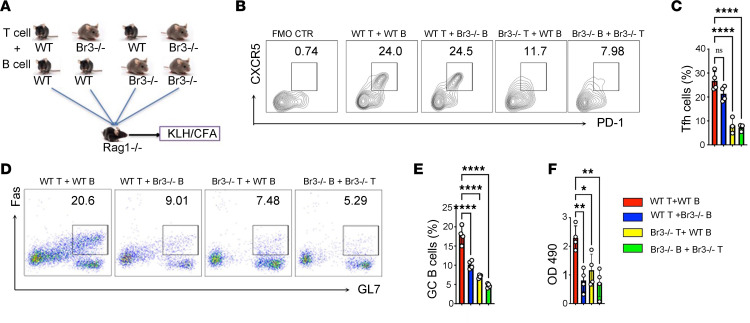
BAFF engagement with BR3 on T cells is required for generation of Tfh cells in vivo. (**A**) Purified T and B cells were sorted from *Br3^–/–^* and WT mice separately and then cotransferred to *Rag1^–/–^* recipient mice as indicated (*n* = 4 per group). Forty-eight hours later, the recipient mice were immunized with KLH/CFA. (**B** and **C**) Tfh cells were analyzed by flow cytometry after 10 days immunization. (**D** and **E**) GC B cells were determined by flow cytometry. (**F**) ELISA for KLH-specific IgG in serum are presented. Data are presented as mean ± SEM. Data are representative of 3 independent experiments. *P* values were calculated by 1-way ANOVA (**D**–**F**) with a Tukey’s multiple comparison test. *P < 0.05, **P < 0.01, ****P < 0.0001.

To document the importance of BAFF engagement with BR3 on T cells for Tfh cell differentiation, we performed adoptive transfer studies. Purified B and T cells from either WT or *Br3*^–/–^ donor mice were transferred into *Rag1*^–/–^ mice (38), which were then immunized with KLH/CFA (Figure 4A). As expected, mice reconstituted with WT T cells and WT B cells developed strong Tfh cell responses. Also as expected, mice reconstituted with *Br3*^–/–^ T cells and *Br3*^–/–^ B cells developed weak Tfh responses. Strikingly, mice reconstituted with WT T cells and *Br3*^–/–^ B cells also developed strong Tfh cell responses, whereas mice reconstituted with *Br3*^–/–^ T cells and WT B cells also developed weak Tfh responses (Figure 4, B and C). That is, the determinant of a strong Tfh response in response to immunization is the presence of BR3 (to engage BAFF) on T cells rather than on B cells.

In contrast, generation of a robust GC B cell response required BR3 expression on both T and B cells. Strong GC B cells developed only in mice reconstituted with WT T cells and WT B cells. In mice reconstituted with *Br3*^–/–^ T cells or *Br3*^–/–^ B cells (or both), the GC B cells responses were weak (Figure 4, D and E). Despite the absence of BR3^+^ Tfh cells, residual GC formation still occurs, likely due to CFA-induced IL-6 and IL-21 production, which promotes Tfh cell formation. After transferring naive T cells into *Rag1*^–/–^ mice followed by CFA immunization, we observed that CFA indeed induced IL-6 and IL-21 production but did not significantly increase Tfh cells (Supplemental Figure 1, A and B; supplemental material available online with this article; https://doi.org/10.1172/jci.insight.183400DS1). Notably, PD-1 expression was markedly elevated (Supplemental Figure 1B). These data suggest that alternative pathways may contribute to induce GC responses.

In alignment with these findings, the levels of KLH-specific IgG antibodies were significantly lower in *Rag1*^–/–^ mice reconstituted with *Br3*^–^ T cells, *Br3*^–^ B cells, or both, compared with the mice reconstituted with WT T cells and WT B cells (Figure 4F). These data indicate that BAFF regulates Tfh cell development by directly interacting with BR3 on T cells, whereas GC B cell development requires interactions between BAFF and BR3 on both B cells and T cells.

### Tfh function in vivo does not require BAFF.

**Figure 5:**
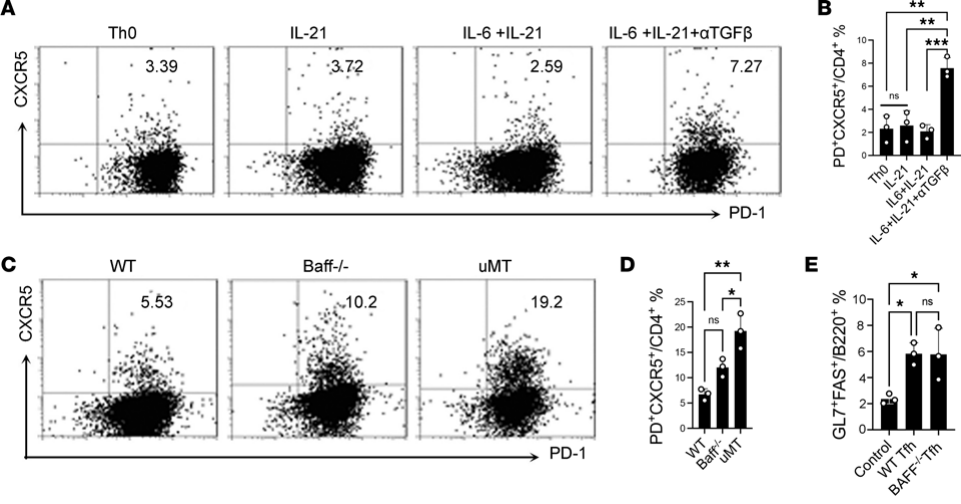
Tfh function in vivo does not require BAFF. (**A** and **B**) CD4^+^ naive T cells were stimulated by coating them in anti-CD3/CD28 in the presence of cytokine as indicated. (**C** and **D**) CD4^+^ naive T cell were isolated from *Baff^–/–^*, μMT, and WT mice and cultured under Tfh condition for 3 days. The represented flow data (**C**) and the statistical data are shown (**D**). (**E**) *Baff^–/–^* and WT Tfh-like cells were transferred into *Baff^–/–^*/Bcl-2 Tg recipient mice separately. These recipient mice were immunized with KLH/CFA, and the GC B cells were determined. Data are presented as mean ± SEM. Data are representative of 3 independent experiments. *P* values were calculated by 1-way ANOVA with a Tukey’s multiple comparison test. *P < 0.05, **P < 0.01, ***P < 0.001.

Purified naive CD4^+^ T cells can be induced into Tfh-like cells in the presence of IL-6, IL-21, and anti–TGF-β, and these Tfh-like cells are capable of effectively inducing GC reactions in vivo (29). Consistent with this, we found that IL-21 alone or the combination of IL-21 and IL-6 was incapable of driving anti-CD3–stimulated CD4^+^ naive T cells into Tfh-like cells, whereas the combination of IL-6, IL-21, and anti–TGF-β successfully did so (Figure 5, A and B). Interestingly, CD4^+^ naive T cells isolated from *Baff*^–/–^ and μMT mice were readily induced into Tfh-like cells (Figure 5, C and D), indicating that generation of such cells does not strictly depend on either BAFF or B cells but can occur, at least in vitro, under BAFF- and B cell–independent conditions.

Moreover, the in vivo function of Tfh-like cells does not require BAFF. WT or *Baff*^–/–^ Tfh-like cells derived from WT or *Baff*^–/–^ hosts were adoptively transferred into *Baff*^–/–^.*Bcl2^Tg^* mice, and these reconstituted mice were immunized with KLH/CFA. Regardless of the source of the transferred Tfh-like cells, the resulting in vivo GC responses were similar (Figure 5E). That is, once Tfh cells are induced, BAFF is dispensable for their function.

### BAFF directly binds to BR3 receptor to enhance Tfh cell differentiation via the NF-κB signaling pathway.

**Figure 6:**
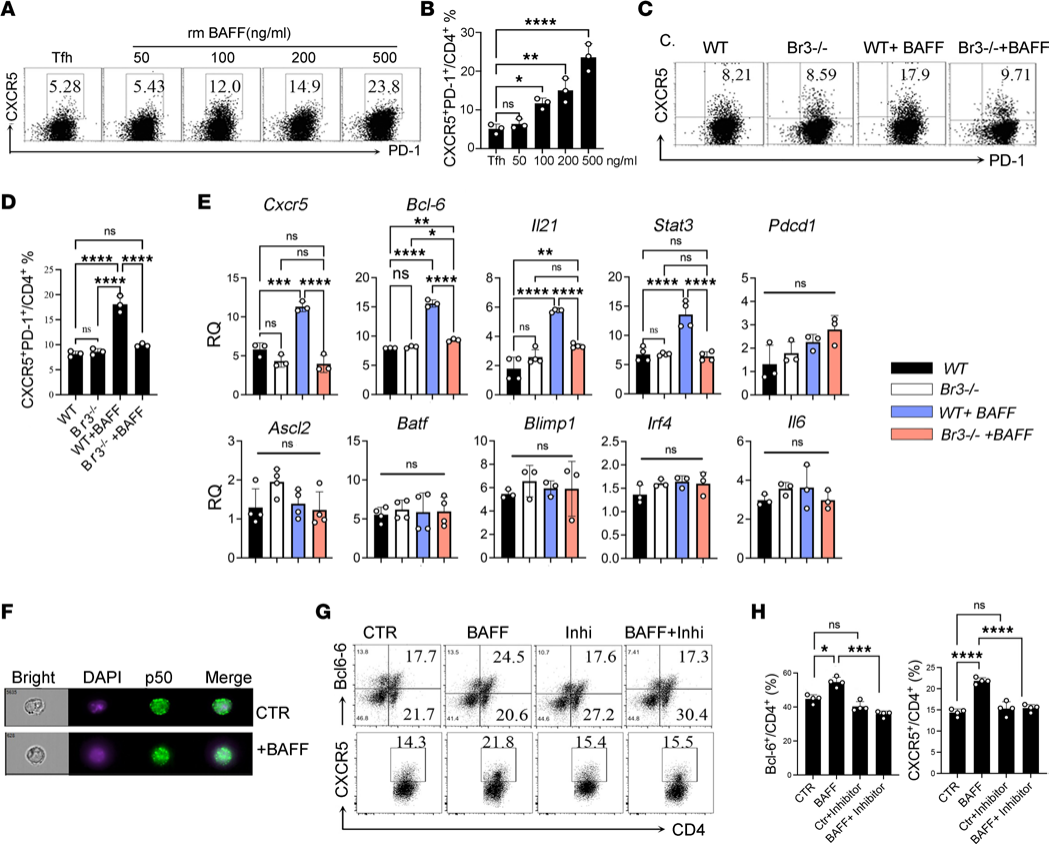
BAFF directly binds to BR3 receptor to enhance Tfh cell differentiation via the NF- κB signaling pathway. CD4^+^ naive T cells were purified from WT mice and cultured under Tfh condition. (**A** and **B**) BAFF enhances Tfh induction in vitro. (**C**–**E**) Naive CD4^+^ T cells from WT and Br3^–/–^ mice were cultured under Tfh conditions with or without BAFF. Tfh frequency (**C** and **D**) and Tfh-related gene (**E**) expression were determined. (**F**) NF-κB p50 distribution was analyzed. (**G** and **H**) NF-κB inhibitor (Inhi) reversed the BAFF-induced BCL-6 and CXCR5 expression. Data are presented as mean ± SEM. Data are representative of 4 independent experiments for **H** and 3 independent experiments for the other panels. *P* values were calculated by 1-way ANOVA (**B** and **D**) with a Tukey’s multiple comparison test. *P < 0.05, **P < 0.01, ***P < 0.001, ****P < 0.0001.

Although BAFF is not absolutely required for generation of Tfh-like cells in vitro or for function of Tfh cells in vivo, BAFF does augment differentiation of naive CD4^+^ T cells into Tfh cells. When WT naive CD4^+^ T cells were cultured under Tfh cell–generating conditions, addition of exogenous BAFF enhanced the response in a dose-dependent manner (Figure 6, A and B). When the experiment was repeated with naive CD4^+^ T cells from *Br3*^–/–^ mice, the effects of BAFF on Tfh cell generation were greatly diminished (Figure 6, C and D). In these cultures, expression of *Bcl6*, *Cxcr5*, *Il21*, and *Stat3* mRNA was enhanced by BAFF, whereas expression of other Tfh cell–related genes, including *Ascl2*, *Batf*, *Irf4*, *Pdcd1*, *Blimp1*, and *Il6*, remained unchanged (Figure 6E). These data confirmed that some, but not all, elements of BAFF-driven induction of Tfh cells are dependent on BR3 expression.

The prosurvival effect of BAFF on B cell survival is via NF-κB signaling (39). To establish that NF-κB signaling is also involved in BAFF-driven Tfh cell generation, we documented p50 translocation from the plasma to the nucleus following BAFF (Figure 6F). An NF-κB signaling inhibitor blocked the effect of BAFF on Bcl-6 and CXCR5 expression (Figure 6, G and H). In summary, our findings indicate that BAFF interaction with BR3 enhances Tfh cell development in an NF-κB signaling–dependent manner.

## Discussion

Although autoantibody-associated autoimmune diseases are highly heterogeneous, most exhibit increased levels of BAFF and excessive Tfh responses (40). While numerous studies have confirmed the role of BAFF/APRIL in maintaining B cell homeostasis, investigation into the role of BAFF/APRIL and its receptors in Tfh cell differentiation had heretofore been limited. Based on considerable experimental evidence, this is the first report to our knowledge that demonstrates the vital role for engagement by BAFF of BR3 on T cells in Tfh cell development.

First, antigen-driven Tfh cell, GC, and antibody responses are greatly blunted in *Baff*^–/–^, but not *April*^–/–^, mice. This suggested a vital role in these responses for BR3 engagement, since APRIL binds to each of the BAFF receptors but does not bind to BR3 (7). The vital role for BR3 was confirmed through experiments in *Taci*^–/–^, *Bcma*^–/–^, and *Br3*^–/–^ mice, in which antigen-driven Tfh cell and antibody responses were blunted in *Br3*^–/–^ mice but not in *Taci*^–/–^ or *Bcma*^–/–^ mice. In vitro experiments identified activation of the NF-κB pathway as being crucial to Tfh cell generation, leading to upregulation of several Tfh cell–associated genes, including *Bcl6*. That is, not only does BAFF activate NF-κB signaling and increase expression of BCL-6 in GC B cells, but it does so as well in T cells (41, 42).

It should be noted that, in addition to BR3, Tfh cells also express BCMA. However, engagement of BCMA not only does not promote Tfh cell generation, but likely inhibits it. Indeed, loss of BCMA from Tfh cells facilitates signaling through BR3 and promotes, rather than diminishes, Tfh cell accumulation in autoimmune-prone B6.*Nba2* mice (26).

Second, the effect of BAFF on Tfh cell generation can be uncoupled from its effect on B cells. Although BAFF/BR3 signaling plays a critical role in transmitting survival signals in B cells and is essential for their differentiation from immature to mature stages. The absence or mutation of BR3 leads to a marked reduction in mature B cells, underscoring its pivotal role in promoting B cell survival. Additionally, BR3’s role contrasts with that of other receptors, such as TACI, which acts as a negative regulator in this process, and BCMA, which is primarily involved in the later stages of B cell differentiation (43). Antigen-driven Tfh cell and GC responses were blunted in *Baff*^–/–^.*Bcl2^Tg^* mice despite the abundance of B cells in these mice. Indeed, adoptive transfer experiments documented that BAFF engagement of BR3 on T cells, rather than on B cells, was essential for antigen-driven Tfh cell generation. In contrast, antigen-driven GC responses required BAFF engagement of BR3 on both T cells and B cells.

Third, in vitro studies indicated that Tfh cell generation can occur through a BAFF-independent pathway. Experiments failed to identify an in vivo, BAFF-independent pathway for antigen-driven Tfh cell generation in *Baff*^–/–^.*Bcl2^Tg^* mice, but it remains possible that BAFF-independent Tfh cell generation could ensue in vivo in other hosts. Indeed, features of systemic lupus erythematosus (SLE), including fatal nephritis, developed in BAFF-deficient NZM.*Baff*^−/−^.*Bcl2^Tg^* mice (44). However, the role, if any, for Tfh cells in this murine model remains uncertain, since Tfh cells were not analyzed in this study.

Fourth, and potentially of great clinical significance, Tfh cells, once generated, do not require BAFF to execute their function. That is, even in a BAFF-sufficient host who had developed a BAFF-dependent Tfh cell response, subsequent neutralization of BAFF would not block the effector function of the Tfh cells already generated. This inability to block Tfh cell function may have contributed to the substantial percentages of patients with SLE who did not experience a meaningful clinical response in clinical trials with the BAFF antagonist, belimumab (45, 46). Since BAFF not only is vital to B cell survival and differentiation and to Tfh cell generation but also contributes to T regulatory cell responses (47), the net effects of pharmacologic neutralization of BAFF in autoimmune disorders (both B cell–associated and non–B cell–associated) may not be easily predictable and will require careful assessment prior to clinical implementation.

### Conclusion.

Our findings establish that BAFF not only has direct effects on B cells, but it also has direct effects on Tfh cell differentiation via engagement of BR3 on T cells, which collectively promote GC formation and production of high-affinity antibodies. The specific engagement of BAFF with BR3 on T cells initiates a cascade of events via NF-κB signaling that culminates in the upregulation of genes quintessential for Tfh cell identity, such as Bcl-6 and Cxcr5. This dual influence of BAFF on both T cell differentiation and B cell maturation provides a mechanistic basis for the observed therapeutic benefits of BAFF inhibition in autoimmune conditions, highlighting its potential as a target for immunomodulatory therapies.

## Methods

### Sex as a biological variable.

Our study examined male and female animals, and similar findings are reported for both sexes.

### Mice.

*Baff*^–/–^ (2), *Baff* Tg (48), *April*^–/–^ (5), *Bcl2^Tg^* (49), μMT (50), *Bcma*^–/–^ (2), *Taci*^–/–^ (51), *Br3*^–/–^ (33), and *Rag1*^–/–^ (52) mice were bred onto the C57BL/6 background, and *Baff*^–/–^.*Bcl2^Tg^* and *Baff*^–/–^.*April*^–/–^ mice were generated by intercrossing the relevant parental strains. All mice were maintained in specific pathogen–free facilities of the University of Southern California, Pennsylvania State University, and Shanghai Jiao Tong University. All animals were handled according to IACUC and facility guidelines.

### Flow cytometry and antibodies.

Cells were labeled with directly conjugated antibodies, including Percp-cy5.5-CD4 (GK1.5), BV510-PD-1 (29F.1A12), Biotin-CXCR5 (L138D7), PE-Biotin (1D4-C5), FITC-CD3 (17A2), PE-CD19 (1D3), APC-B220 (RA3-6B2), PE-Dazzle 594-Fas (DX2), AF700-GL7 (GL7), and APC-Bcl-6 (7D1) from Biolegend. Dead cells were excluded using Fixable Viability Dye eFluor780 (Thermo Fisher). Flow cytometry analysis was performed as described previously (53) using a Celester Flow Cytometer (BD Bioscience) and BD Fortessa. Data were analyzed using Flowjo software.

### Amnis ImageStream.

To visualize NF-κB p50 (Thermo Fisher) localization in CD4^+^ T cells, cells were stained with anti–NF-κB p50 followed by PE-conjugated goat anti-rabbit IgG secondary antibody. Approximately 5 × 10^3^ cells were acquired by Amnis ImageStream (Merck), and the data were processed using Ideas 4.0 software (Merck).

### KLH immunization.

The mice were subcutaneously immunized at the base of the tail with 50 μL of a solution containing 0.5 mg/mL KLH emulsified in 0.5 mg/mL CFA. Ten days after immunization, mice were sacrificed and analyzed individually.

### ELISA.

Sera from immunized KLH immunized mice were serially diluted 10-fold and added to 96-well plates (Nunc) precoated with 10 μg/mL KLH protein. Antigen-specific antibodies were detected using HRP-conjugated anti-mouse IgG (Thermo Fisher, 50400) and HRP-conjugated anti-mouse IgG2a (Thermo Fisher, 50420). Data were presented as an OD value at 450 nm (OD450).

### Immunofluorescence.

Spleen cells were fixed for 1 hour at 4°C in a solution containing 4% paraformaldehyde and 10% sucrose in PBS and incubated overnight in 30% sucrose. Samples were embedded in optimum cutting temperature compound and then cryosectioned. All the samples were stained and mounted using Prolong Gold anti-fade Reagents (Invitrogen) and examined using a Leica confocal system as previously reported (21). The antibodies used were Biotin-PNA (B-1075, Vector Laboratories), PE-IgD (11-26c, BioLegend), PE-CD4 (GK1.5), and Alexa Fluor 488 streptavidin (Biolegend). No positive signal was detected in the controls where only the secondary antibody (anti-Biotin) was used without the primary antibody.

### Cell purification and differentiation in vitro.

CD4^+^CD62L^+^ naive T cells were sorted from enriched CD4^+^ T cells (52). For Tfh cell induction in vitro, naive CD4^+^ T cells were stimulated with mouse anti-CD3 (5 μg/mL, 145-2C11, Biolegend), anti-CD28 (5 μg/mL, 37.51, Biolegend), 20 ng/mL IL-6 (R&D Systems), 20 ng/mL IL-21 (R&D Systems), 10 μg/mL anti–IL-4 (11B11, Biolegend), 10 μg/mL anti–IFN-γ (XMG1.2, Biolegend), and 20 μg/mL anti–TGF-β (1D11, R&D Systems) in the presence of mitomycin-pretreated antigen presenting cells for 4 days (30).

### Lymphocyte adoptive transfer.

B220^+^ B cells and CD90.2^+^ T cells were sorted from the splenocytes of WT or *Br3*^–/–^ mice. B cells from WT or *Br3*^–/–^ (5 × 10^6^) were mixed with T cells from either WT or *Br3*^–/–^ (5 × 10^6^) and then injected via the tail vein into *Rag1^–/–^* mice. Sixteen hours after cell transfer, the recipient mice were immunized with KLH emulsified in CFA.

### Quantitative real-time PCR.

CD4^+^ T cells were purified from the harvested cells, and total RNA was extracted using Trizol Reagent (Thermo Fisher, 15596026CN) according to the manufacturer’s instructions. The quality and quantity of RNA were measured with Nanodrop (Thermo Fisher). cDNA was synthesized using the RT-master Mix (RR036, TAKARA) and amplified by quantitative reverse transcription PCR reagents (RR430, TAKARA) in the ABI 7900 Prism system. The data were analyzed using the relative gene expression method and normalized to HPRT Ct values in the samples. Each sample was measured in triplicate. The primer sequences used for the PCR amplification were as follows: HPRT forward: 5′-GGTGGTCTCCTCTGACTTCAAC-3′, HPRT reverse: 5′-GTTGCTGTAGCCAAATTCGTTG-3′; Cxcr5 forward: 5′-TAAGGCGGTATTCATGCCTGT-3′, Cxcr5 reverse: 5′-GCTACTGCGAGGTGGAACA-3′; Bcl-6 forward: 5′-CGGGTCCCTGGAAACATCG-3′, Bcl-6 reverse: 5′-TCGGGAGTATTCGGTTTGAAT-3′; Stat3 forward: 5′-AGAACCTCCAGGACGACTTTG-3′, Stat3 reverse: 5′-TCACAATGCTTCTCCGCATCT-3′; Il21 forward: 5′-CAGTCTGGCAACAACTCCCAA-3′, Il21 reverse: 5′-GTTCGTCATAAGCTGAGTCCC-3′; Pdcd1 forward: 5′-ACCCTGGTCATTCACTTGGG-3′, Pdcd1 reverse: 5′-CATTTGCTCCCTCTGACACTG-3′; Ascl2 forward: 5′-AAGCACACCTTGACTGGTACG-3′, Ascl2 reverse: 5′-AAGTGGACGTTTGCACCTTCA-3′; Batf forward: 5′-AGCGAGCTGCTGACTGAGA-3′, Batf reverse: 5′-CGCCTTACTGGTGTGCTTCT-3′; Blimp1 forward: 5′-TTCTCTTGGAAAAACGTGTG-3′, Blimp1 reverse: 5′-GGAGCCGGAGCTAGACTTG-3′; Irf4 forward: 5′-TCCGACAGTGGTTGATCGAC-3′, Irf4 reverse: 5′-CCTCACGATTGTAGTCCTGCTT-3′; Il6 forward: 5′-CTGCAAGAGACTTCCATCCAG-3′, Il6 reverse: 5′-AGTGGTATAGACAGGTCTGTTGG-3′.

### Statistics.

Statistical analysis was performed using GraphPad Prism. Data were analyzed by 2-tailed Student’s *t* test in the case of 2 groups and 1-way ANOVA analysis for 3 and more groups. Data are presented as mean ± SEM if not otherwise indicated. A value of *P* less than 0.05 was considered to be statistically significant.

### Study approval.

The animal use was approved by the IACUC at Guangdong Provincial People’s Hospital (Dongguan, China) and The Ohio State University (Columbus, Ohio, USA).

### Data availability.

All supporting data values associated with the main manuscript and supplemental materials, including individual data points shown in graphs and values behind any reported means, are provided in the Supporting Data Values file.

## Author contributions

Conceptualization was provided by SGZ and WS. Methodology was provided by SGZ, WS, YC, MC, YL, QL, JW, WL, YFP, LL, JC, RL, JZ, Y Xiong, and Youqiu Xue. Investigation was performed by SGZ, WS, YC, MC, YL, QL, JW, WL, YFP, LL, JC, RL, JZ, Y Xiong, and Youqiu Xue. Funding was acquired by SGZ, YC, and YL. Project administration was provided by SGZ and WS. Supervision was provided by SGZ and WS. The original draft was written by YC. The manuscript was reviewed and edited by SGZ and WS.

## Supplementary Material

Supplemental data

Supporting data values

## Version 1 09/26/2024

In-Press Preview

## Version 2 11/08/2024

Electronic publication
