# Supplementary material for: BAFF promotes follicular helper T cell development and germinal center formation through BR3 signal
Source: JCI Insight. 2024 Nov 8;9(21):e183400. doi: 10.1172/jci.insight.183400 (PMC11601555; doi:10.1172/jci.insight.183400)
Supplement: Supplemental data [file jciinsight-9-183400-s128.pdf]

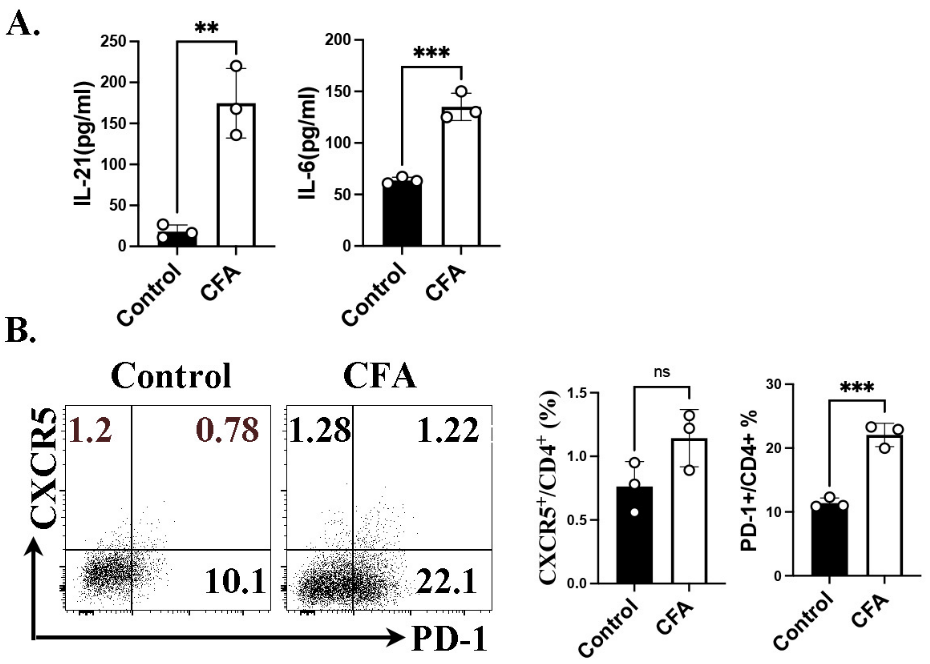

Supplement Figure 1. Stimulation with CFA enhances the IL-6 and IL-21 production. Purified Naïve T cells were transferred to Rag1$^{-/-}$ recipient mice (n=3 per group), 7 days later, the recipient mice were immunized with CFA. **(A)** Serum concentrations of IL-6 and IL-21 were measured by ELISA**.** (**B**) The expression levels of PD-1 and CXCR5 on CD4^{+} T cells were assessed using flow cytometry after 7 days immunization. *p* values were calculated by Student's t-test. **p*< 0.05, ***p*< 0.01, ****p*< 0.001, *****p*< 0.0001, ns, not significant.
